# Cross-sectoral digital planning Drinking Water-Urban Heat-Housing Provision indicators dataset

**DOI:** 10.1016/j.dib.2025.112310

**Published:** 2025-11-22

**Authors:** Iván Cardenas-Leon, Pirouz Nourian, Mila Koeva, Karin Pfeffer

**Affiliations:** Department of Urban and Regional Planning and Geo-Information Management, Faculty of Geo-Information Science and Earth Observation (ITC), University of Twente, Hallenweg 8, Enschede, 7522 NH, Overijssel, Netherlands

**Keywords:** Urban indicators, Integrated planning, Data-driven policy, Spatial planning, Environmental monitoring, Infrastructure indicators, Open urban data, Literature review

## Abstract

This dataset paper presents a comprehensive compilation of indicators covering three major domains: drinking water supply, urban heat, and housing provision. The data were systematically collected from a wide range of peer-reviewed papers to ensure a robust foundation for both academic research and practical policy analysis. The dataset includes a total of 280 indicators, each categorized and described. The indicators are organized into a relational database format, which allows for easy access and manipulation. The dataset was collected to facilitate cross-sectoral analysis, enabling researchers and practitioners to explore the interconnections between these critical areas of urban planning. The key indicators are described with clear definitions, calculations, and provenance information. Additionally, the dataset is organized in a relational SQL database, allowing users to perform advanced queries, join operations, and custom analyses. Potential applications span a range of research areas, including urban planning, environmental impact assessment, public health, and socio-economic studies.

Specifications Table

**Table:** 

Subject	Social Sciences
Specific subject area	Planning and Development; Drinking water; Urban Heat; Housing provision
Type of data	Table (Relational). Processed data. SQL schema
Data collection	Data was collected through a Structured Rapid Review following the Preferred Reporting Items for Systematic Reviews and Meta-Analyses (PRISMA)[14] to identify the key impact indicators measured in the planning activities concerning water supply, urban heat stress, and provision of housing.
Data source location	The raw data sources are listed in the GitHub and Zenodo data repositories. Data was collected from multiple journals using Scopus, Web of Science and Geobase databases.
Data accessibility	Repository name: **Zenodo** Data identification number: 10.5281/zenodo.17464468 Direct URL to data: https://github.com/ivan-cardenas/DigitalPlanning_Indicators Read the README.md file. Data can be read using both MS Access software (.accdb file) and any other database reader (.sql file). Data is also available as a collection of CSV files. See interactive version at https://ivan-cardenas.github.io/PapersData/DataPaper.html
Related research article	None

## 1 Value of the Data

• **Supports cross-sectoral planning**: The dataset provides a comprehensive overview of the indicators used in the water supply, urban heat stress, and housing provision planning sectors. This can help researchers and practitioners identify synergies and trade-offs between these sectors.
• **Facilitates evidence-based decision-making:** By providing a structured overview of key urban indicators, if data is available in the local context, the dataset can be used to support evidence-based decision-making in urban planning and development.
• **Aids in the digital transformation of urban planning**: The dataset can be used to identify policy targets for the implementation of digital planning tools such as Urban Digital Twins, which can help improve the efficiency and effectiveness of urban planning processes.
• **Helps identify research and data gaps:** This dataset can help researchers identify gaps in the existing literature and inform future research directions in the fields of water supply, urban heat stress, and housing provision planning. Likewise, it gives a list of data required for research, then can help focus data collection efforts.
• **Facilitates knowledge sharing:** The dataset can be used to promote knowledge sharing and collaboration among researchers, practitioners, and policymakers in the fields of water supply, urban heat stress, and housing provision planning.
• **Provides a structure for performing a rapid systematic review:** The dataset can be used as a reference for researchers and practitioners looking to perform a rapid systematic review for data extraction and transform the data into a structured, shareable, and reusable format.

## 2 Background

Given the complexity of urban and regional planning and the many stakeholders involved, **planners have increasingly adopted a fragmented, sector-specific approach** rather than an integrated cross-sector perspective [17]. **Effective spatial planning**, however, demands a comprehensive understanding of **interrelationships, tradeoffs, and overlapping interactions** across social, environmental, and economic dimensions and **across planning sectors** [10,19]. To significantly enhance stakeholder collaboration and drive data-informed planning and policymaking through comprehensive urban insights, cities should adopt digital planning tools that leverage technological advancements for a responsible, **digitally enabled planning** process [6,20].

This single-sector approach limits the potential for integrated planning and decision-making, as it does not consider the interdependencies and interactions between different sectors [5,12]. Proposing a digital planning tool that can be used to support cross-sectoral planning and decision making requires the identification of the key indicators and user requirements of each sector to measure the performance, challenges, and critical aspects of planning for an individual sector [8,9].

In this sense, the dataset collected here aims to have a comprehensive list of indicators that are important for the planning and policy making of water supply, measurement of urban heat stress, and housing provision. These three topics were selected to try to understand how the European housing crisis [18], and its subsequent need for new homes, can affect or be affected by the drinking water availability and its provision, and how the new constructions could increase the urban heat island effect. Likewise, the constructions could have an impact on the water cycle and therefore affect the availability of water for provision to residents. A whole cycle that can benefit from a digitally integrated planning approach.

## 3 Data Description

This dataset has two main items: A relational database with a set of indicators and a bibliographic information file. The database contains a list of indicators related to the three sectors: drinking water supply, urban heat stress, and housing provision. The bibliographic information file contains the references of the papers used to extract the indicators.

### 3.1 Relational database

The database [1] is structured in a Microsoft Access database format (.accdb), and a copy of it is also provided in SQL format (.sql) for compatibility with other relational database management systems (Postgres, MySql, SQLite), and in Comma Separated Values files (.csv) for accessibility to users not familiar with databases.

**Fig. 1:**
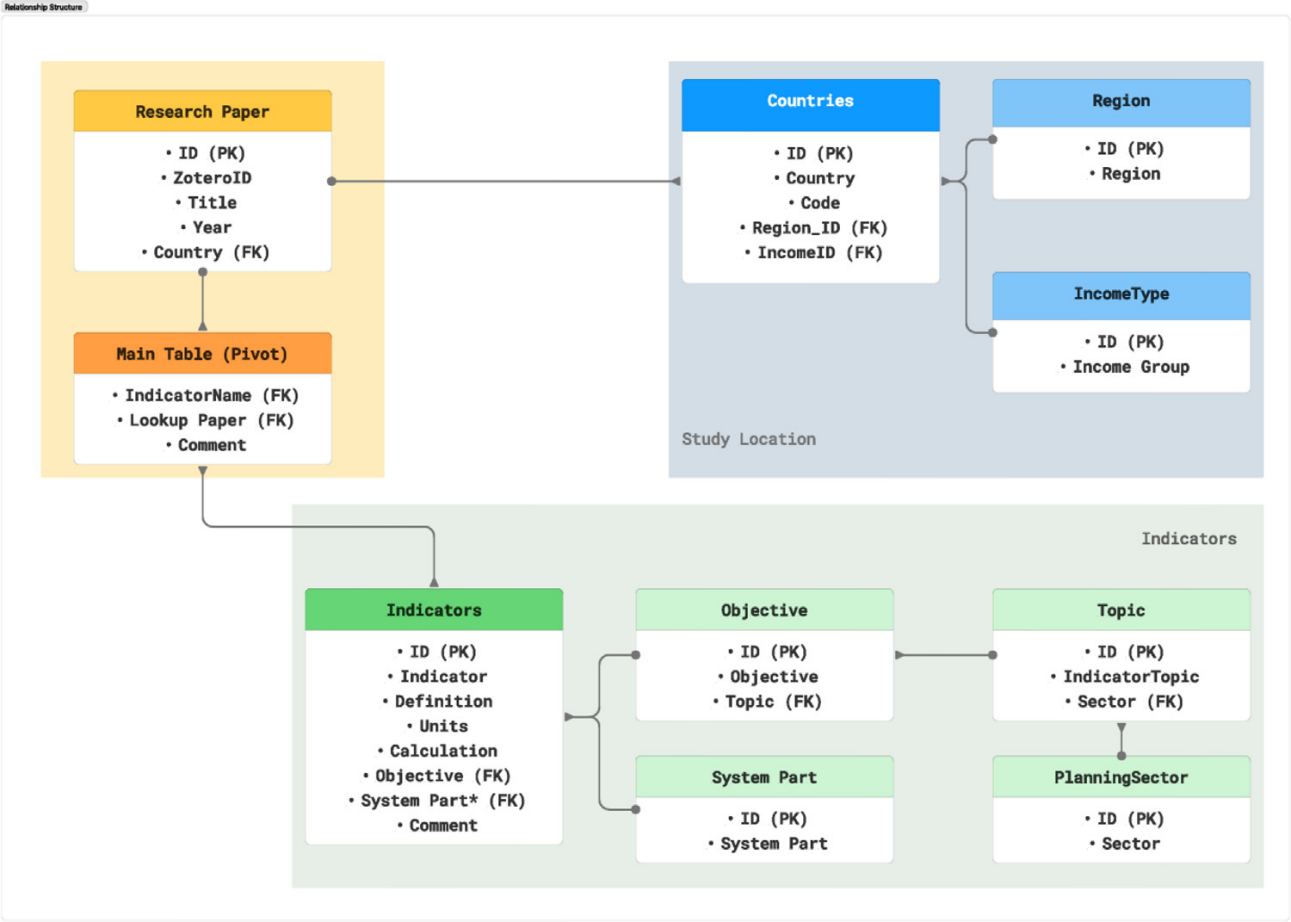
Database schema. The figure shows the conceptual relational structure of the database. Each table has a Sequential ID.

All versions contain identical data and structure, ensuring accessibility for users with different software preferences. The database is organized into several tables, each representing a specific aspect of the data. The tables are described Table 1. Each table is related to the others through a set of primary and foreign keys based on the schema presented in Fig. 1.

**Table 1:** Description of the tables in the database.

Table Name	Description
Countries	List of countries and ISO codes (222 records)
Region	Seven world regions plus worldwide studies (8 records^1^)
IncomeType	Four income categories + ‘Not Defined’ for multi-country studies (5 records^2^)
Research Paper	Bibliographic list of papers (560 records)
Objective	Measurement objectives (17 records)
Topic	Topics related to objectives (7 records)
System Part	Water supply system parts (19 records)
Indicator	Indicator details extracted from research papers (280 records)
Main Table	Pivot linking indicators to papers (1608 records)

1 Includes a record for Worldwide studies and other for continental or subcontinental studies.

2 Includes a ’Not Defined’ record for multi-country studies and for Venezuela.

### 3.2 Data structure

**Fig. 2:**
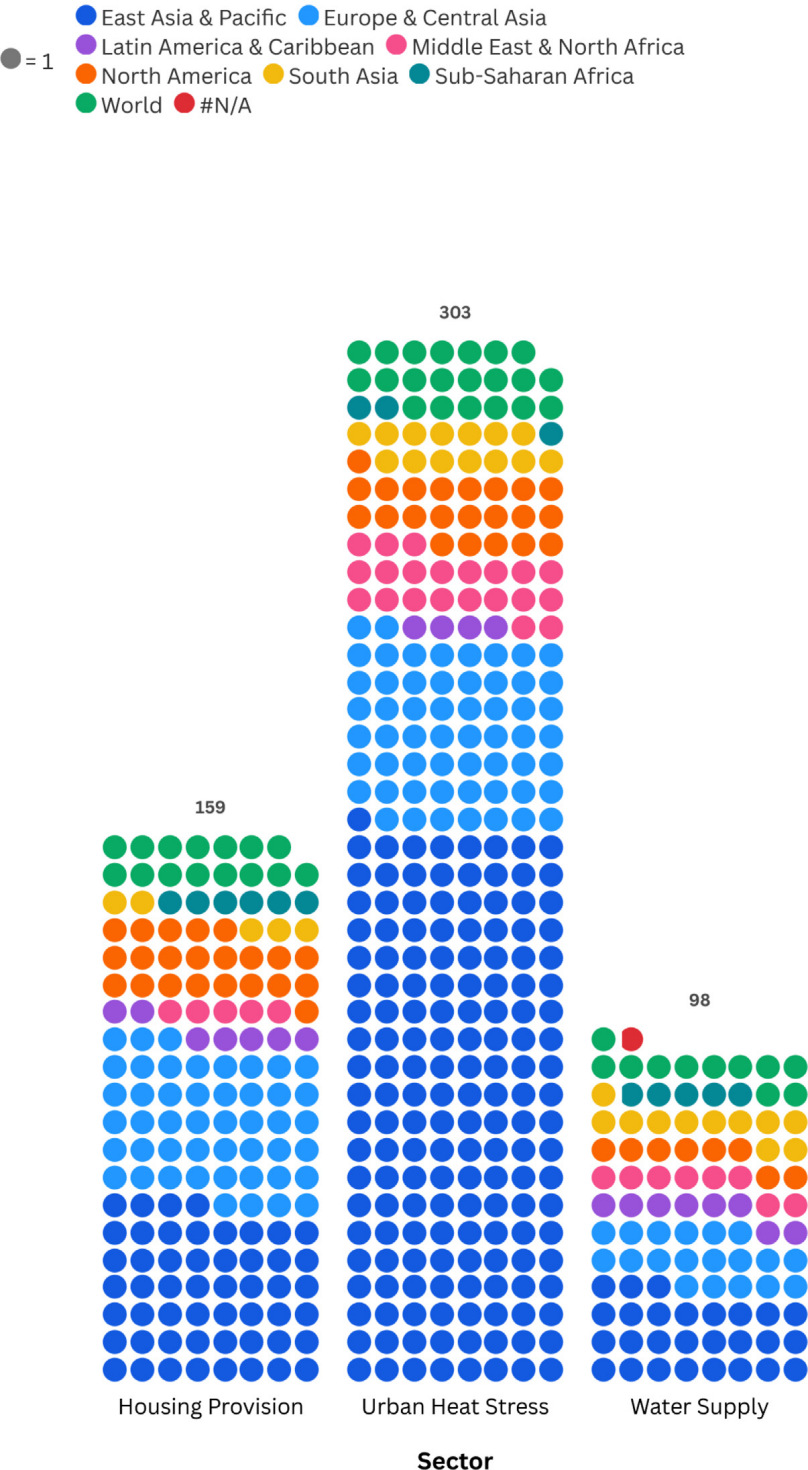
Distribution of the studies per region. See the Interactive version for detailed country distribution \#N/A category corresponds to a virtual setup, not linked to a case study in the real world.

There is a total of 560 research papers used to extract the indicators. From them, 98 are for drinking water supply, 303 for urban heat stress, and 159 for housing provision. Each paper can have one or more indicators related to it, but only one country of study is related to each research paper. Fig. 2 shows the distribution of the studies per region. There are 46 studies that are done across different countries and world regions (multi-country), while 13 are done in different countries but on the same continent. The description of the data type for each attribute of the tables is shown in Table 2. Each table has a Sequential ID for indexing and acting as a Parent Key (PK), and can connect to other tables via a Foreign Key (FK).

**Table 2:** Description of the data type for each attribute of the tables.

Table	Attribute	Description	Data Type
Countries	CountryCode Country Code RegionID IncomeID	ISO code of the country. Name of the country. ISO code of the country. Name of the region. Name of the income type.	Text Text Text Integer (FK) Integer (FK)
Region	ID Region	Sequential ID of the region (PK) Name of the region.	Integer (PK) Text
IncomeType	ID Income Group	Sequential ID of the Income types Name of the income type.	Integer (PK) Text
Research Paper	ID ZoteroID Title Year Country	Sequential ID of the research paper. Biblatex ID of research paper - connects to the bibliographic data. Title of the research paper. Year of publication. Name of the country.	Integer (PK) Text Text Integer (FK)
Objective	ID Objective Topic	Sequential ID of the objective. Name of the objective of the measurement. Name of the topic that encompasses the objectives.	Integer (PK) Text Integer (FK)
Topic	ID Topic Sector	Sequential ID of the topic. Name of the topic that encompasses the objectives. Name of the Planning sector that encompasses the topics	Integer (PK) Text Integer (FK)
	ID PlaSector	Sequential ID of the sector Name of the Planning sector - Water supply, Urban Heat, Housing Provision.	Integer (PK) Text
Indicators	ID Indicator Definition Units Calculation System Part ^1^ Objective Topic Comment	Sequential ID of the indicator. Name of the indicator. Definition of the indicator. Units on which the indicator is measured. Equation used to calculate the indicator Name of the system part of the water supply system. Name of the objective of the measurement. Name of the topic that encompasses the objectives. Comments about the indicator.	Integer (PK) Text Text Text Text Integer (FK) Integer (FK) Integer (FK) Text
Main Table	IndidicatorName Lookup Paper Comment	Name of the indicator. Biblatex ID of research paper - connects to the Bibliographic data. Comments about the indicator inside the research paper.	Integer (FK) Integer (FK) Text

^1^ This attribute is only for the Water Supply indicators, as the system is comprised of several parts that are clearly distinguishable: e.g., pumps, pipes, tanks, valves, and meters.

**Fig. 3:**
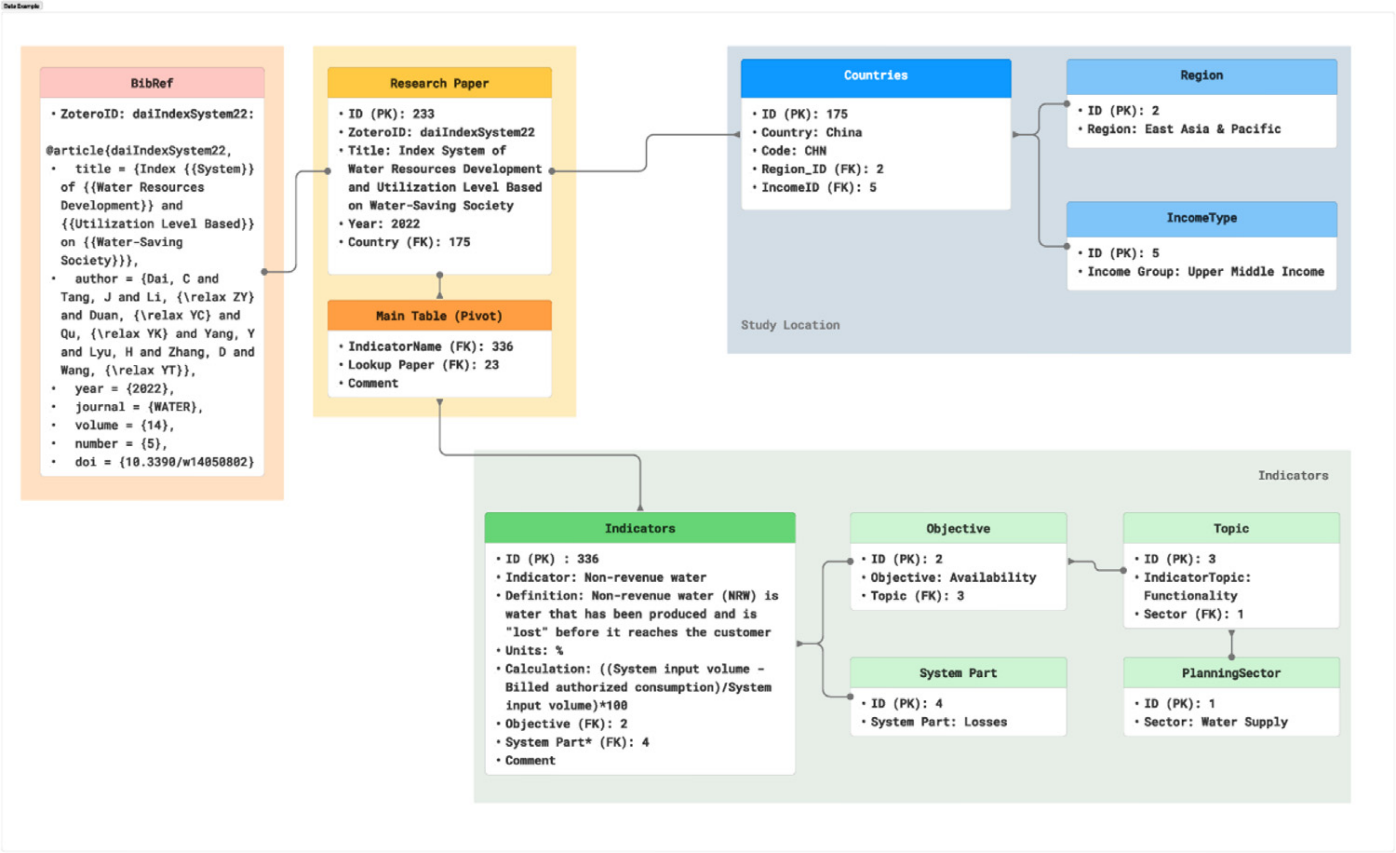
Example of data structure in the database.

As an example, Fig. 3 shows the structure of the database with the identified indicator. The figure illustrates how the different tables are connected from the study location and country income type to the details of the indicator and the objective of its measurement. Likewise, it shows how the Research paper table connects with the bibliographic reference.

### 3.3 Indicators dataset

#### 3.3.1 Drinking water supply indicators

**Fig. 4:**
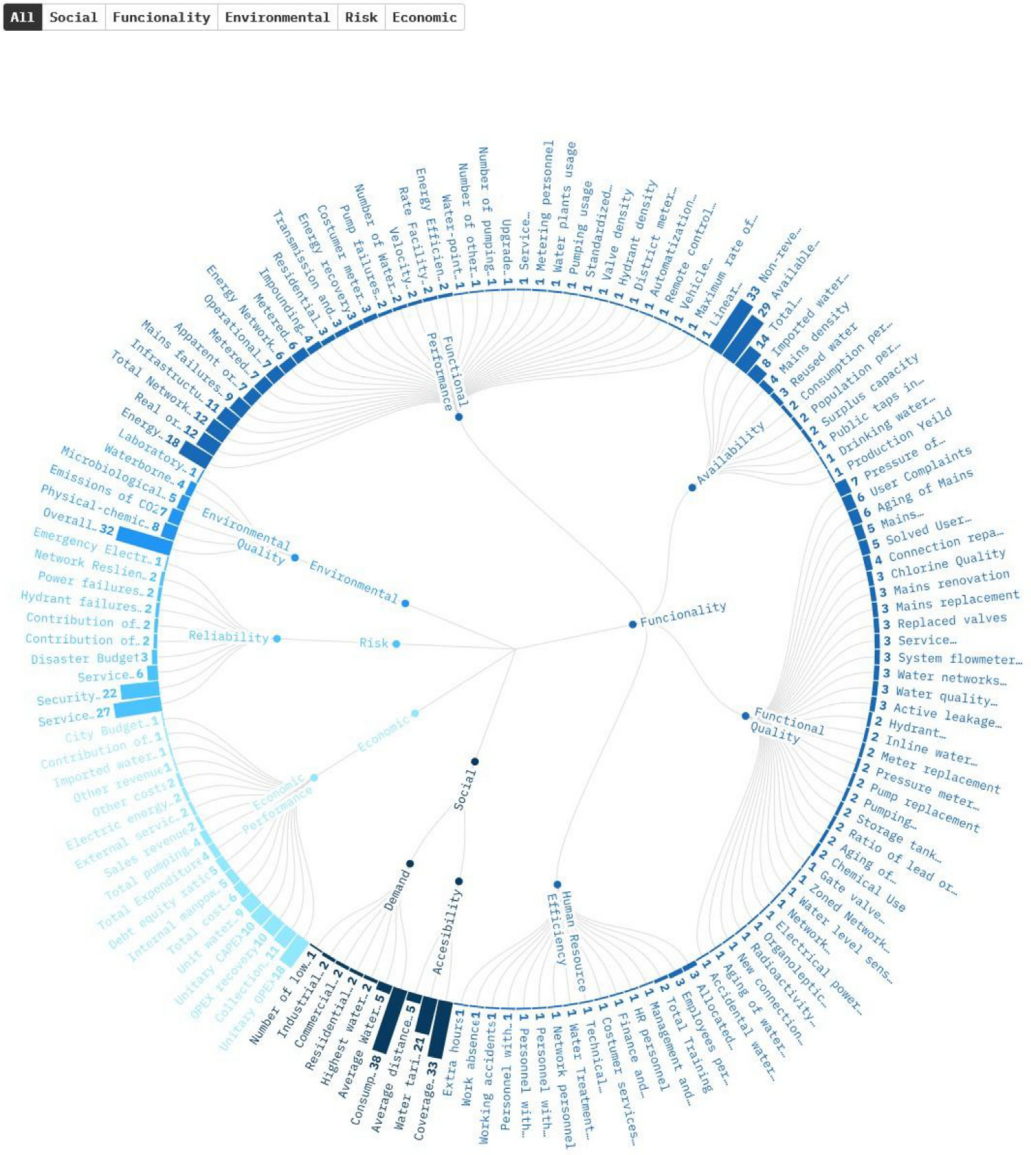
Water Supply Indicators in literature. See the Interactive version and tabular summary for details of each indicator and filtering.

The 98 studies included in this rapid review had 145 unique water supply planning and management indicators, organized into five topics and nine objectives. As papers used several indicators in the same publication, a total of 680 records were found and organized by frequency. It is important to highlight that four studies focus on indicators that can be used worldwide and not in country-specific contexts [2,7,13], being the OECD document and Allegre et al. (2017) cited frequently by other authors as the key reference for water supply security planning. 131 records out of the 680 (19.24 %) represent these worldwide indicators. Meanwhile, only 29 records were focused on three Low-income countries: Eritrea, Ethiopia, and the Republic of Yemen. Most records are for high-income countries, with 171 records from 29 studies. China (19.42 %) and India (10.68 %) are the countries with the most studies in this sector of the review. The top five most frequent indicators are Consumption per capita, non-revenue water, Coverage of water supply, Overall Water Quality, and Available Fresh Water Per Capita. Fig. 4 shows the distribution of the indicators by topic and objective.

### 3.4 Urban heat stress indicators

**Fig. 5:**
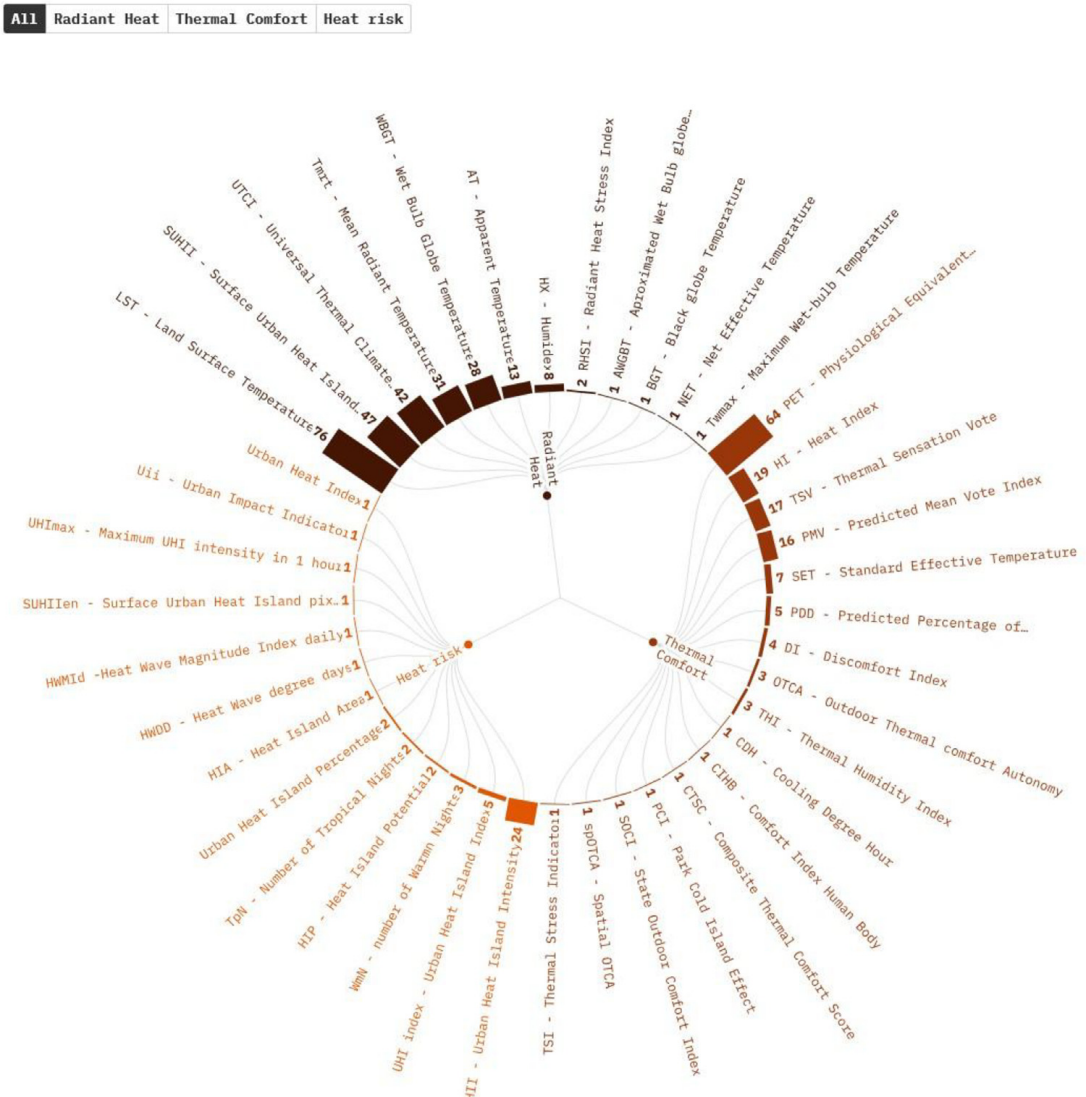
Urban heat stress Indicators in literature. See the Interactive version and tabular summary for details of each indicator and filtering.

The 303 studies included in this rapid review had 41 unique urban heat stress indicators. As papers used several indicators in the same publication, a total of 435 records were found and organized by frequency. Of these 435, 27 records (6.21 %) were used to measure urban heat stress on a global scale. Only one study focused on a Low income country: Ethiopia. Most records are for upper-middle-income countries, with 209 records from 161 studies. China (42.24 %) and the United States (5.94 %) are the countries with the most studies in this sector. Fig. 5 shows the distribution of the indicators by topic and objective. The top five most frequent indicators are Land Surface Temperature (LST), Physiological Equivalent Temperature index (PET), Surface Urban Heat Island Intensity (SUHII), Universal Thermal Climate Index (UTCI), and Mean Radiant Temperature (Tmrt).

### 3.5 Housing provision indicators

**Fig. 6:**
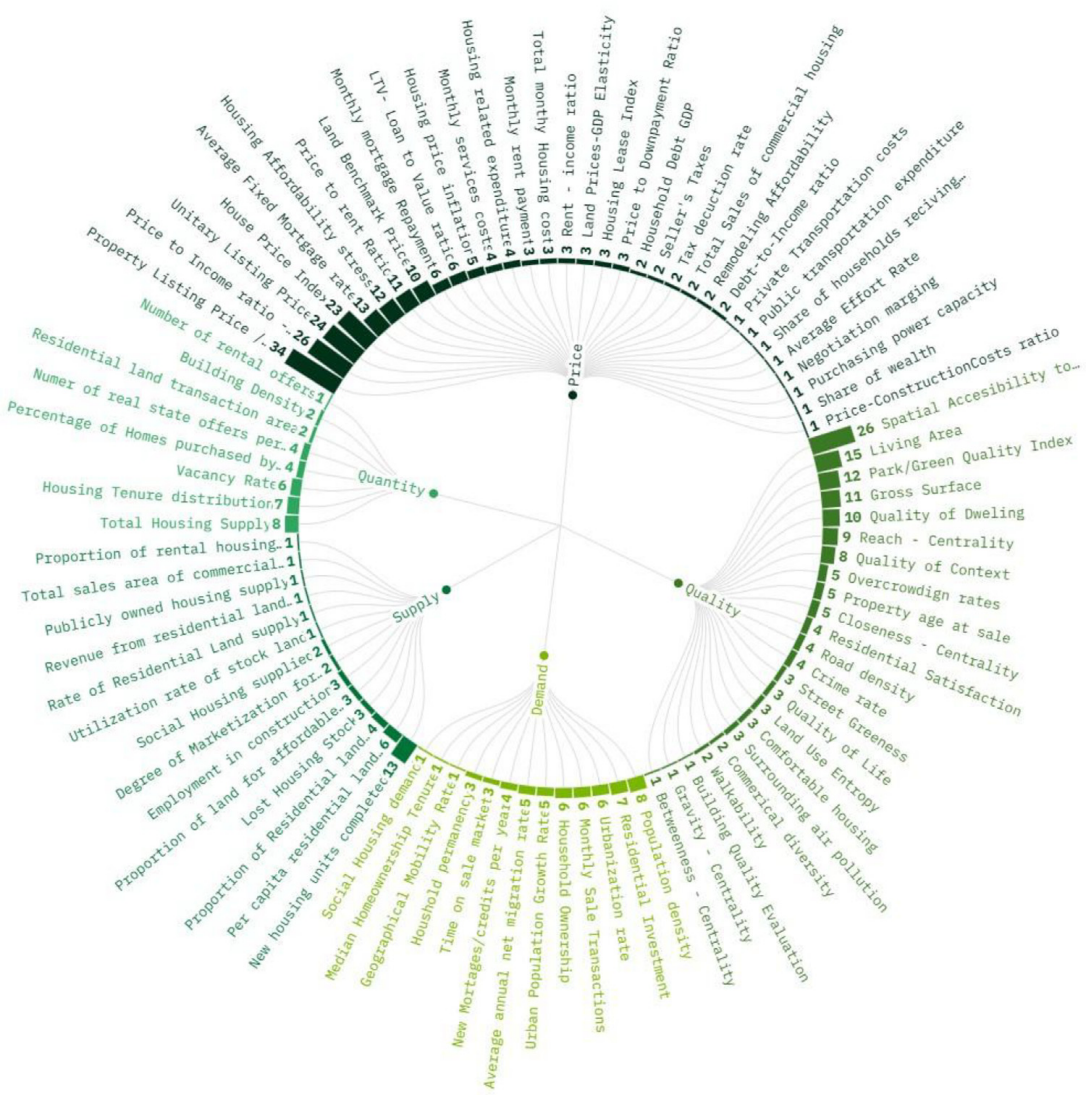
Housing Provision Indicators in literature. See the Interactive version and tabular summary for details of each indicator and filtering.

The 159 studies included in the rapid review had 93 unique housing provision indicators. As papers used several indicators in the same publication, a total of 488 records were found and organized by frequency. Of these 488, 64 records (7.06 %) were used to measure Housing provision across different cities and countries. Only one study focused on a Low-income country: Ethiopia. Most records are for High-income countries (42.3 %), with 199 records from 73 studies. China (21.38 %) and the United States (13.84 %) are the countries with the most studies in this sector of the review. Fig. 6 shows the distribution of the indicators by topic and objective. The top five most frequent indicators are Property Listing Price or Sale Price, Price to Income ratio (affordability), Spatial Accessibility to Facilities, Unitary Listing Price, and House Price Index.

### 3.6 Bibliographic data

The bibliographic data is stored in a .ris file that can be imported into any bibliographic manager. For this dataset, we collected and organized the research papers using Zotero. The citation key of each item connects to the database via the Paper table and the ZoteroID attribute.

## 4 Experimental Design, Materials and Methods

A rapid review of research papers was made to identify the key indicators measured in the water supply planning and performance assessment. For water supply security, this review was limited to documents posterior to the literature review made by Rathnayaka et al. [16] where they identify criteria to assess the sustainability of water supply management systems; for urban heat stress, the review was limited to documents after 2019 (last five years), to focus on the most recent approaches to Urban heat and the increasing interest on the topic [11,15]; for housing the review was also limited to documents posterior to 2019 as a result of the changes in the housing market and the impact of COVID-19 on housing policy related to the effects of density building [3]. The rapid review follows the Preferred Reporting Items for Systematic Reviews and Meta-Analyses (PRISMA) statement [14]. The search results on each database were imported into Covidence and Rayyan software to manage the review.

### 4.1 Water supply security

We retrieved 677 documents for this Rapid Review from Scopus, Web of Science, and Geobase databases. Due to the proximity of the topic to waterborne diseases, wastewater management, water physicochemical properties, and water use for agriculture, among others. The terms included in the search were required to be limited, and several exclusion parameters needed to be set in our research query. To filter the results, we used the criteria specified in Table 3. To retrieve previous research, we used the following Scopus query, which we then adjusted to suit the search syntax of the other databases.

**Table 3:** Inclusion and Exclusion Criteria: Water Supply documents.

Inclusion Criteria	Exclusion Criteria
The main focus is on the drinking water supply.	Indicators are not SMT (Specific, Measurable, and Time-bound).
Papers are about human water consumption.	The main focus is on wastewater or stormwater.
Show different indicators to measure performance or goals.	The main focus is on the Water Cycle.
It is about urban water supply.	Paper is about the state of water quality* The document publication date is 2016 or later. The paper is about Water treatment procedures. The paper is about health-related situations: waterborne diseases, infections, and microbiological vectors. Papers are about the agricultural or agronomical use of water. Papers are related to the impact of water on soil properties and erosion. Papers are related to the physicochemical properties of the water. Is in languages other than English, Spanish, or Portuguese.

⁎ This refers to papers that measure water quality and describe their properties, but do not use water quality as an indicator for water supply systems.

TITLE-ABS-KEY ((“water supply” OR “water planning” OR “water infrastructure” OR “potable water” OR “water distribution” OR “water management”) AND (indicators OR metric OR benchmark OR kpi) AND urban AND NOT (rural OR “green space”)) AND NOT KEY (irrigation OR agric* OR agro* OR wastewater OR “waste-water” OR “waste water” OR sewer OR sewage OR stormwater OR “storm water” OR flood* OR *chemic* OR physicochemical OR atmosph* OR waterborne OR infect* OR bacter* OR microbio* OR soil OR erosion OR “green infrastructure”) AND NOT TITLE (irrigation OR agric* OR agro* OR wastewater OR “waste-water” OR “waste water” OR sewer OR sewage OR stormwater OR “storm water” OR flood* OR *chemic* OR physicochemical OR atmosph* OR waterborne OR infect* OR bacter* OR microbio* OR soil OR erosion OR “green infrastructure") AND (LIMIT-TO (DOCTYPE,“ch”) OR LIMIT-TO (DOCTYPE,“ar”) OR LIMIT-TO (DOCTYPE,“re”)) AND (EXCLUDE (SUBJAREA,“MEDI”) OR EXCLUDE (SUBJAREA,“AGRI”) OR EXCLUDE (SUBJAREA,“BIOC”) OR EXCLUDE (SUBJAREA,“IMMU”) OR EXCLUDE (SUBJAREA,“VETE”) OR EXCLUDE (SUBJAREA,“PHAR”) OR EXCLUDE (SUBJAREA,“NURS”) OR EXCLUDE (SUBJAREA,“CHEM”) OR EXCLUDE (SUBJAREA,“MATH”) OR EXCLUDE (SUBJAREA,“PHYS”) OR EXCLUDE (SUBJAREA,“ATE”) OR EXCLUDE (SUBJAREA,“CENG”) OR EXCLUDE (SUBJAREA,“PSYC”) OR EXCLUDE (SUBJAREA,“NEUR”) OR EXCLUDE (SUBJAREA,“HEAL”) OR EXCLUDE (SUBJAREA,“`DENT”) OR EXCLUDE (SUBJAREA,“ENER”)) AND (LIMIT-TO (PUBYEAR,2016)) AND (LIMIT-TO (LANGUAGE,“English”) OR LIMIT-TO (LANGUAGE,“Spanish”) OR LIMIT-TO (LANGUAGE,“Portuguese”)).

**Fig. 7:**
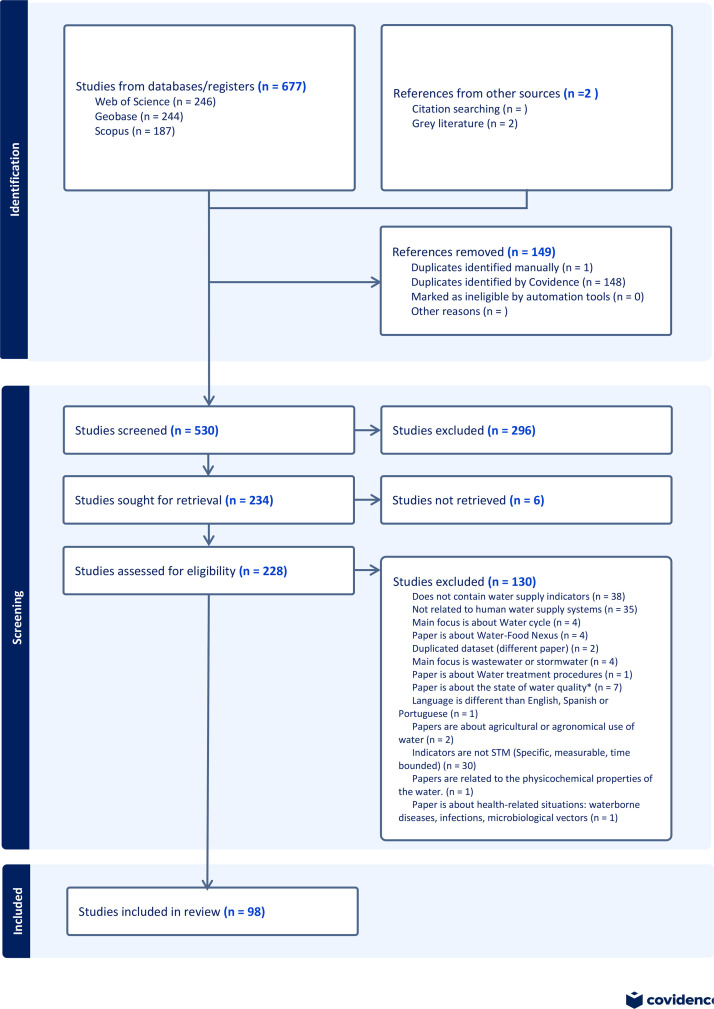
PRISMA flowchart of the Water Supply Rapid Review.

We identified 677 articles in the search. 246 from Web of Science, 244 from Geobase, and 187 from Scopus. We then included two additional references through snowballing, which were highly cited among the retrieved articles. We removed 149 articles as duplicate items in the databases. The remaining 530 articles were screened for title, abstracts, and accessibility. In this way, we excluded 296 articles from the screening of the title and abstract, and six studies were inaccessible. The 228 retrieved articles were assessed based on the criteria from Table 3, which resulted in 126 studies being excluded. This resulted in 98 final studies included in this review, from which the Key indicators were obtained (Fig. 7).

### 4.2 Urban heat stress

We retrieved 1001 documents from Scopus and Web of Science for Urban heat stress. The terms were limited to exclude heat stress related to farm animals or endangered species, as this research focuses on human heat stress. As heat stress could occur in different environments, we filter out documents of heat stress at the workplace or indoors, focusing on outdoor heat (public space) in our search query. The criteria in Table 4 summarize the inclusion and exclusion criteria. The query we used to retrieve previous research was the following:

**Table 4:** Inclusion and Exclusion Criteria urban heat stress documents.

Inclusion Criteria	Exclusion Criteria
The main focus is on the outdoor heat stress.	Indicators are not SMT (Specific, Measurable, and Time-bound).
Show different indicators to measure Urban Heat or urban heat stress.	The main focus is on indoor heat stress
The document publication date is after 2019.	Papers are related to medicine or pharmacology
Documents are Peer-reviewed articles or chapter books	Papers are about animals’ heat stress Paper relates to general Climate Adaptation Plans Relates to occupational Heat Stress Relates to the operation or efficiency of Air conditioning systems Is in languages other than English, Spanish, or Portuguese.

TITLE-ABS-KEY (((“Urban Heat” OR uhi OR “Physiological equivalent temperature” OR “heat stress” OR “Thermal Comfort” OR “Urban Thermal” OR “Urban climate” OR “Urban microclimate”) W/15 (indicators OR metric OR benchmark OR kpi)) AND NOT (indoor OR ice OR “soil quality” OR disease OR biomass OR hvac OR “Air conditioning” livestock OR animal* OR irrigation OR agric* OR agro* OR avia* OR photovoltaic OR pv OR parasite OR host OR coral)) AND (EXCLUDE (SUBJAREA, “MEDI”) OR EXCLUDE (SUBJAREA, “AGRI”) OR EXCLUDE (SUBJAREA, “BIOC”) OR EXCLUDE (SUBJAREA, “IMMU”) OR EXCLUDE (SUBJAREA, “VETE”) OR EXCLUDE (SUBJAREA, “PHAR”) OR EXCLUDE (SUBJAREA, “NURS”) OR EXCLUDE (SUBJAREA, “CHEM”) OR EXCLUDE (SUBJAREA, “MATH”) OR EXCLUDE (SUBJAREA, “PHYS”) OR EXCLUDE (SUBJAREA, “MATE”) OR EXCLUDE (SUBJAREA, “CENG”) OR EXCLUDE (SUBJAREA, “PSYC”) OR EXCLUDE (SUBJAREA, “NEUR”) OR EXCLUDE (SUBJAREA, “HEAL”) OR EXCLUDE (SUBJAREA, “DENT”) OR EXCLUDE (SUBJAREA, “ENER”)) AND (LIMIT-TO (DOCTYPE, “ar”) OR LIMIT-TO (DOCTYPE, “cp”) OR LIMIT-TO (DOCTYPE, “re”)) AND (LIMIT-TO (PUBYEAR, 2019) OR LIMIT-TO (PUBYEAR, 2020) OR LIMIT-TO (PUBYEAR, 2021) OR LIMIT-TO (PUBYEAR, 2022) OR LIMIT-TO (PUBYEAR, 2023) OR LIMIT-TO (PUBYEAR, 2024) OR LIMIT-TO (PUBYEAR, 2025)) AND (LIMIT-TO (LANGUAGE, “English”) OR LIMIT-TO (LANGUAGE, “Spanish”) OR LIMIT-TO (LANGUAGE, “Portuguese”)) AND (EXCLUDE (EXACTKEYWORD, “Office Buildings”) OR EXCLUDE (EXACTKEYWORD, “Indoor Air”) OR EXCLUDE (EXACTKEYWORD, “Indoor Air Quality”) OR EXCLUDE (EXACTKEYWORD, “Indoor Environment”) OR EXCLUDE (EXACTKEYWORD, “Animal”) OR EXCLUDE (EXACTKEYWORD, “Intelligent Buildings”) OR EXCLUDE (EXACTKEYWORD, “Nonhuman”) OR EXCLUDE (EXACTKEYWORD, “HVAC”)).

**Fig. 8:**
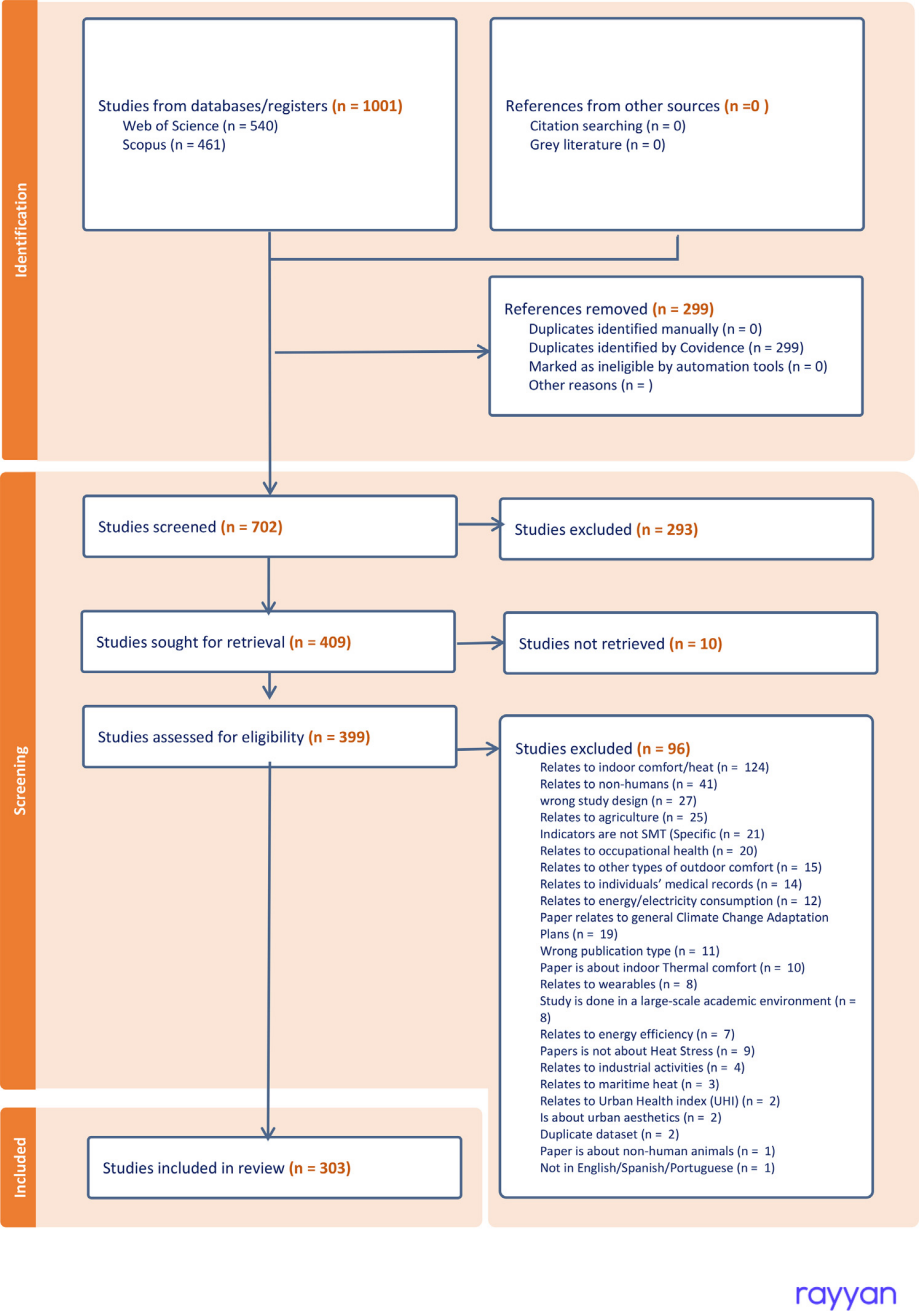
PRISMA flowchart of the urban heat stress Rapid Review.

We identified 1001 articles in the search. 540 from Web of Science, and 461 from Scopus. We removed 299 articles as duplicate items in the databases. The remaining 702 articles were screened for title, abstracts, and accessibility. In this way, we excluded 293 articles from the screening title and abstract, and ten studies were inaccessible. The 399 retrieved articles were assessed based on the criteria from Table 3, which resulted in 96 studies being excluded. This resulted in 303 final studies included in this review, from which the Key indicators were obtained (Fig. 8).

### 4.3 Housing provision

We retrieved 522 documents from Scopus and Web of Science for Housing Provision in the five main groups proposed by Angel et al. [4] in the Housing Indicators program: *price, quantity, quality, demand* and *supply*. The terms used in the query exclude results relating to household appliances, home respiratory diseases, and nursing homes. The criteria in Table 5 summarize this planning sector’s inclusion and exclusion criteria. The query we used to retrieve previous research was the following:

**Table 5:** Inclusion and Exclusion Criteria: Housing provision documents.

Inclusion Criteria	Exclusion Criteria
The main focus is on Housing demand and supply.	Indicators are not SMT (Specific, Measurable, and Time-bound).
Show different indicators to measure Housing price, housing quantity, housing quality, housing demand, and/or housing supply	The main focus is not on Housing
The document publication date is after 2019.	Papers are related to medicine or nursing homes
Documents are Peer-reviewed articles or chapter books	Papers are about housing energy demand Research focuses on Macroeconomic indicators or the Financial Market Research relates to tourism Research focuses on the House of Quality Methodology Is in languages other than English, Spanish, or Portuguese.

TITLE-ABS-KEY(((Home OR Hous* OR residential) W/5 (affordability OR Demand OR Supply OR price OR quality)) W/15 (indicators OR metric OR benchmark OR kpi)) AND NOT (“Domestic appliances” OR respiratory OR “Care Services” OR diseases OR agric* OR agro* OR avia*) AND NOT (SUBJAREA(MEDI) OR SUBJAREA(AGRI) OR SUBJAREA(BIOC) OR SUBJAREA(IMMU) OR SUBJAREA(VETE) OR SUBJAREA(PHAR) OR SUBJAREA(NURS) OR SUBJAREA(CHEM) OR SUBJAREA(MATH) OR SUBJAREA(PHYS) OR SUBJAREA(MATE) OR SUBJAREA(CENG) OR SUBJAREA(PSYC) OR SUBJAREA(NEUR) OR SUBJAREA(HEAL) OR SUBJAREA(DENT) OR SUBJAREA(ENER)) AND (LIMIT-TO (DOCTYPE,“ar”) OR LIMIT-TO (DOCTYPE,“cp”) OR LIMIT-TO (DOCTYPE,“re”)) AND (LIMIT-TO (PUBYEAR,2019) OR LIMIT-TO (PUBYEAR,2020) OR LIMIT-TO (PUBYEAR,2021) OR LIMIT-TO (PUBYEAR,2022) OR LIMIT-TO (PUBYEAR,2023) OR LIMIT-TO (PUBYEAR,2024) OR LIMIT-TO (PUBYEAR,2025)) AND (LIMIT-TO (LANGUAGE,“English”) OR LIMIT-TO (LANGUAGE,“Spanish”) OR LIMIT-TO (LANGUAGE,“Portuguese”)).

**Fig. 9:**
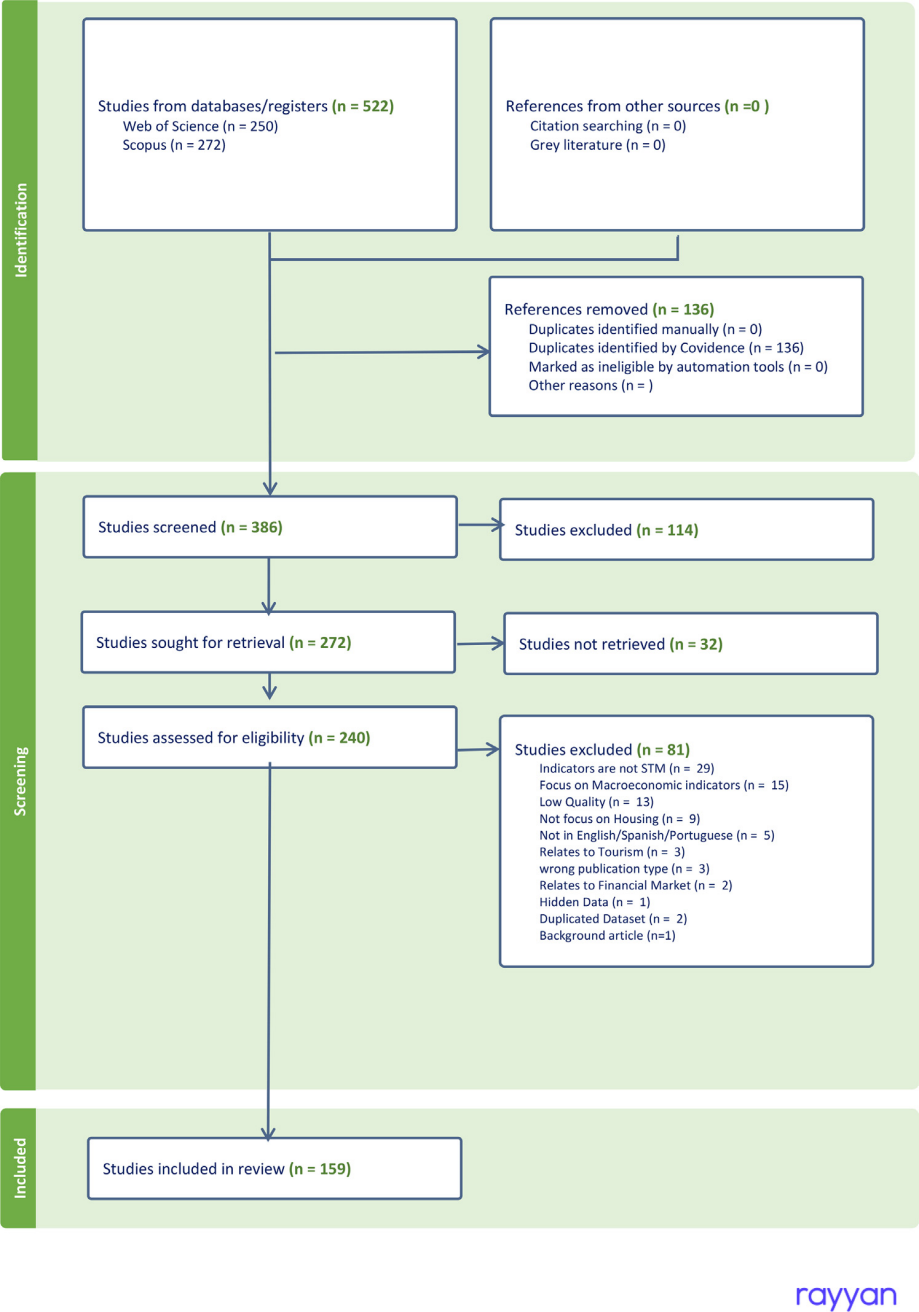
PRISMA flowchart of the Housing Provision Rapid Review.

From the search query for Housing provision, we identified 522 articles. 272 from Scopus and 250 from Web of Science. We removed 136 articles as they are duplicate items in both databases. The remaining 386 articles were screened for titles and abstracts. In this way, we excluded the other 114 articles. It was not possible to retrieve 32 papers due to accessibility restrictions. The remaining 240 articles were assessed based on the criteria from Table 5, which resulted in 81 studies being excluded. This resulted in 159 final studies in this review for full-text assessment (Fig. 9).

### 4.4 Updating database

Researchers and practitioners are invited to collaborate and submit pull requests via GitHub to continually update the repository, either in the current reviewed planning sectors or in other sectors that highlight cross-sectoral interdependencies. This can be done by cloning the GitHub repository or directly editing the CSV files, following the data structure presented in this paper, which is also available as a JSON file in the repository. The repository contains an automatic executable script that validates the structure of the new data via a Python script executed when the pull request is performed. We suggest following the rapid literature review approach and the presented database structure to establish a common framework for storing indicators used in urban planning.

## Limitations

This review was limited to studies published in languages spoken by the first author—English, Spanish, and Portuguese—which may have excluded relevant literature in other languages. The extracted indicators are not exhaustive and may not capture all dimensions of water supply, urban heat stress, and housing provision planning. Additionally, references from GeoBase were excluded from the urban heat stress and housing literature, as the number of records retrieved from the other sources was large and deemed a manageable size to identify the most used indicators over the past five years. Future research is encouraged to expand the database by including indicators and sources not covered by the current search strategy.

## Ethics Statement

The authors confirm that the current work does not involve human subjects, animal experiments, or any data collected from social media platforms.

## Credit Author Statement

**IvánCardenas-Leon:** Conceptualization, Data curation, Formal analysis, Methodology, Validation, Visualization, Writing - original draft. **Pirouz Nourian:** Supervision, Writing - review & editing. **Mila Koeva:** Supervision, Writing - review & editing. **Karin Pfeffer:** Supervision, Writing - review & editing.

## Data Availability

ZenodoCross sectoral digital planning Drinking Water-Urban Heat-Housing Provision indicators dataset (Original data).
